# Cobalt oxide-alumina catalysts for the methane-assisted selective catalytic reduction of SO_2_ to sulfur

**DOI:** 10.1016/j.heliyon.2023.e21269

**Published:** 2023-10-27

**Authors:** Masoud Khani, Seyyed Ebrahim Mousavi, Reza Khalighi, Saeed Abbasizadeh, Hassan Pahlavanzadeh, Habib Ale Ebrahim, Abbas Mozaffari

**Affiliations:** a Faculty of Chemical Engineering, Petrochemical center of Excellency, Amirkabir University of Technology, Tehran, Iran; b Faculty of Chemical Engineering, Tarbiat Modares University, Tehran, Iran; c School of Chemical Engineering, College of Engineering, University of Tehran, Tehran, Iran; d Research and Development Unit, Sarcheshmeh Copper Complex, Kerman, Iran

**Keywords:** Cobalt oxide, Alumina, Catalyst, Selective catalytic reduction, SO_2_, Methane

## Abstract

Preventing emission of pollutants in any kind, is a way to protect global environment. The objective of this study is to develop cobalt catalysts supported on alumina for the conversion of the toxic gas SO_2_ into elemental sulfur using methane. Although several useful catalysts have been proposed, there is still a need to synthesize a catalyst with a high sulfur yield that is also persistent during on-stream stability. To this end, four different catalysts were prepared using the wet impregnation technique, with Co_3_O_4_ content ranging from 0 to 15 wt%. Catalytic activity tests were carried out at atmospheric pressure and temperatures ranging from 550 to 800 °C. The Al_2_O_3_–Co (15 %) catalyst exhibited superior performance, with a sulfur yield of 98.1 % at 750 °C. The catalytic stability of the best catalyst was examined using a 20 h on-stream stability test under the optimized conditions including an SO_2_/CH_4_ molar feed ratio of 2 at 750 °C. The structural changes of the used catalyst after the stability test were investigated using XRD and TPO analyses. It was revealed that sulfidation of Co_3_O_4_ after a short while, results in decreasing the sulfur yield from 98.1 % to 89.8 %.

## Highlights

• The best catalyst, Al_2_O_3_–Co (15 %), achieved a sulfur yield of 98.1 %.
• After the on-stream stability test, the major formed species were identified as Co_3_O_4_, CoS, and CoS_2_.
• It was observed that the Co_3_O_4_ species were mainly responsible for the catalytic reduction of SO_2_ with CH_4_.

## 1 Introduction

The question of whether it is easier to address human misbehaviors and preserve the current planet or to explore extraterrestrial life remains a topic of debate. Every year, numerous industries release thousands of tons of hazardous gases, including NO_x_, CO, and SO_2_, into the atmosphere due to the absence of effective post-treatment processes. The emission of SO_2_, in particular, can result in a range of adverse consequences, including chronic respiratory issues and even fatalities in humans, as well as the destruction of fish populations via decreasing water pH and acid rain, which can damage soil, plants, and structures. Therefore, it is crucial to implement flue gas desulfurization (FGD) techniques to mitigate SO_2_ emissions from flue gas sources. FGD methods can be classified into two groups: throwaway and regenerative processes [1,2].

The major throwaway process for small amounts of SO_2_ emission is “Lime Sorption” while for high flow rates of SO_2_, using this method would result in a vast useless material which must be discarded [3,4]. The regenerative techniques offer better alternatives in these cases, especially for metal roasting plants. Catalytic conversion of SO_2_ into sulfuric acid and elemental sulfur are the most familiar regenerative methods. Sulfuric acid production would be favored when there is a good demand close to the plant due to its high corrosive nature and difficulties in storage and transportation. On the other hand, sulfur production is more interesting and promising with regards to its easier storage and transport [5].

For selective catalytic reduction (SCR) of SO_2_ to elemental sulfur, several reducing agents have been applied including CO [[6], [7], [8]], H_2_ [[9], [10], [11]], CH_4_ and syngas (CO + H_2_) [12]. Although mild operating conditions while implementing CO and H_2_, their production costs are notably high. Considering CH_4_ as the reducing agent, its major drawback is higher operating temperatures in comparison with H_2_ and CO. However, lower price and better accessibility make CH_4_ a better choice, particularly for the countries with large natural gas reserves (such as Iran, Russia and etc.).

Investigating catalytic reduction of SO_2_ with CH_4_ has been initiated with alumina catalysts. Helstrom et al. [13] used a commercial bauxite sulfur recovery catalyst to propose a simple kinetic model at temperatures between 500 and 650 ᵒC. Borbin et al. [14,15] studied SCR of SO_2_ with CH_4_ in the presence of an industrial activated alumina at the temperature range of 732–831 ᵒC and SO_2_/CH_4_ ratios of 0.5, 0.66 and 1 when CH_4_ is in excess. Also, a similar work has been done by Sarlis et al. [16] in a temperature range of 650–750 ᵒC with SO_2_/CH_4_ ratios of 0.5–2.5 using activated alumina. Yermakova et al. [17] evaluated another kinetic model for activated alumina in a wider temperature range of 650–850 ᵒC using a metallurgical gas mixture with CH_4_/(SO_2_+O_2_) ratios between 0.6 and 1.17. MoO_3_, MoS_2_ and binary molybdenum-cobalt oxides supported on alumina and activated carbon [[18], [19], [20], [21], [22], [23]] were other fields of interest due to their good activity. Additionally, high SO_2_ reduction activity of ceria via CH_4_ even with low active surface area led researchers to synthesized binary and ternary ceria-based catalysts [[24], [25], [26], [27], [28]]. Shikina et al. [29] prepared ferromanganese nodules supported on alumina and Ca-montmorillonite with a reliable catalytic activity for SCR of SO_2_ with CH_4_.

Cobalt catalysts supported on alumina exhibited a good performance for many reactions such as Fischer–Tropsch process [[30], [31], [32]], Biginelli reaction [33], dry reforming of methane [34] and steam reforming of ethanol [35]. Also, Yu et al. [36] synthesized cobalt oxide catalysts on different supports of silica, zeolite and alumina towards SCR of SO_2_ with methane. They reported alumina as the most active support overall while considering only 30 wt% for Co_3_O_4_ species on the supports. In our previous works, nickel oxide and molybdenum oxide catalysts on alumina were studied for SCR of SO_2_ by methane [37,38]. In this work, cobalt oxide catalysts with four weight percent of 0, 5, 10 and 15 supported on γ-alumina were prepared and characterized through BET, XRD, XRF and FESEM. Next, their catalytic activity towards SO_2_ reduction by CH_4_ were examined and compared. Finally, for the best catalyst, the effect of feed gas SO_2_/CH_4_ ratio and gas hourly space velocity (GHSV), besides on-stream stability (as an important parameter in industrial applications) were investigated carefully [39].

## 2 Experimental section

### 2.1 Catalyst preparation

Herein, the wet impregnation technique was used for catalyst preparation [40]. In the initial stage, three distinct aqueous solutions of cobalt nitrate (Co(NO_3_)_2_·6H_2_O, sourced from Merck) were prepared with molar concentrations of 0.1, 0.2, and 0.3. To each of these solutions, 10 g of a commercial γ-Al_2_O_3_ support purchased from Ardakan Industrial Ceramics Company was added. The γ-Al_2_O_3_ support exhibited a spherical shape with an average diameter of 3 mm. Following the addition of the support, the resulting materials were subjected to a mixing process for 1 h using a rotary evaporator, after which the excess water was gradually removed at a temperature of 80 °C. Subsequently, the samples were further dried overnight at 120 °C in an oven. Ultimately, they underwent a calcination process at 550 °C for a duration of 4 h. As a result, four different weight percentages (0 %, 5 %, 10 %, and 15 %) of Co_3_O_4_ species were introduced onto the γ-Al_2_O_3_ support, denoted as Al_2_O_3_, Al_2_O_3_–Co (5 %), Al_2_O_3_–Co(10 %), and Al_2_O_3_–Co(15 %), respectively.

### 2.2 Catalyst characterization

Using nitrogen adsorption method, adsorption isotherms, pore size distributions, and specific surface area of the catalysts were measured with an Autosorb-1MP apparatus of Quantachrome at 77 K. FESEM imaging was done using a Hitachi S-4160 apparatus to compare the surface morphology of the bare and impregnated catalysts.

XRD patterns of the prepared catalysts were obtained for phase detection with a diffractometer (PHILIPS-PW1730) operated at 40 kV and 30 mA with Cu Kα radiation (λ = 1.5406 Å). The XRD device had a scan rate of 3°/min using a step size of 0.05°. Also, in order to evaluate the amount of deposited Co_3_O_4_ species on the support, XRF analysis was performed via PHILIPS PW1410 analyzer.

Temperature programmed oxidation (TPO) test of the used catalyst was carried out with a Rheometric Scientific Instrument device coupled with a mass spectrometer from Leda Mass. The mass spectrometer can detect 12 different gases online with ppm precision after converting peak heights to partial pressure profiles versus time [41].

### 2.3 Catalytic activity tests

**Fig. 1:**
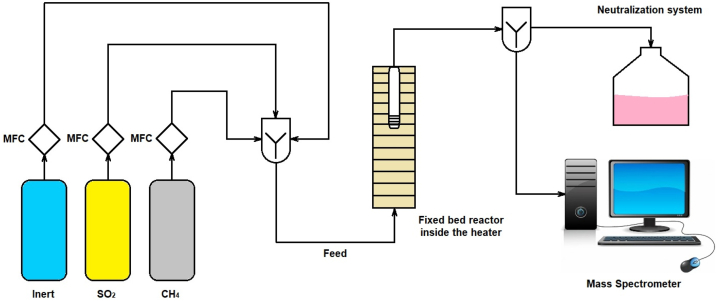
Flow diagram of the reaction test system.

The experiments were carried out in a fixed-bed stainless steel tubular reactor (inner diameter: 1.27 cm, length: 1 m, position of the catalyst bed: 60 cm from the bottom, inner diameter of the catalyst bed: 1 cm) vertically mounted in an electrical furnace. In each test, 1 g of the catalyst was loaded onto the bed inside the reactor. The flow diagram of the experimental setup is shown in Fig. 1.

At first, the reactor was purged by argon gas stream. Then, the system was heated to reach the desired temperature under a mixture of reaction gases of SO_2_, CH_4_ and large amount of argon. Except for the effect of GHSV tests, total volumetric flow of the feed gases was kept at GHSV = 8000 h^−1^. Three mass flow controllers were used to adjust SO_2_, CH_4_ and argon inlet concentrations for the feed flow. The reactor outflow was divided into two streams, one connected to the mass spectrometer (MS) for analysis and the other transferred to an alkaline solution neutralization system.

## 3 Results and discussion

### 3.1 Catalyst characterization

In Fig. S1 results of XRD test for all Al_2_O_3_–Co catalysts are given. According to Fig. S1, by adding Co_3_O_4_ species, alumina major peaks are diminished while Co_3_O_4_ intrinsic peaks begin to rise at 2θ angles of 31.2, 36.8 and 66, distinctively. Also, the presence of Co_3_O_4_ species crystals is fairly clear in Fig. S2 considering Al_2_O_3_ and Al_2_O_3_–Co (5,10 and 15 %) comparatively.

Results of BET surface area and pore size distribution graph together with the amount of loaded Co_3_O_4_ for the prepared catalysts are summarized in Table S1. It was expected that surface area and total pore volume would be smaller for Al_2_O_3_–Co catalysts than the bare alumina resulted from blocking some of the support's pores with cobalt oxide crystals (Table S1). On the contrary, better catalytic activity could be obtained due to the presence of cobalt oxide species as a transition metal oxide active site, similar to our former studies on nickel oxide and molybdenum oxide catalysts [37,38].

Fig. S3 shows BJH pore size distribution of all catalysts as a means of calculating average pore diameter given in Table S1. During introduction of Co_3_O_4_ species to the alumina support, cobalt precursor molecules penetrate into the internal channels and fill finer pores causing Al_2_O_3_–Co catalysts to have smaller total pore volume but a bigger average pore diameter than the alumina. Accordingly, average pore diameter of the alumina support was obtained 43.62 Å, while this parameter increased to 68.95 Å for Al_2_O_3_–Co(15 %).

### 3.2 Catalytic activity tests

#### 3.2.1 Effect of temperature on SO_2_ reduction

The main reaction for SCR of SO_2_ with CH_4_ and the most likely major side reaction between them are presented in equations (1), (2), respectively. While equation (1) produces desired sulfur product, equation (2) generates toxic H_2_S and CO gases.

(1) $${\text{CH}}_{4}+2{\text{SO}}_{2}\to \;2S+{\text{CO}}_{2}+2H_{2}O$$

(2) $$2{\text{CH}}_{4}+{\text{SO}}_{2}\to \;H_{2}S+2\text{CO}+3H_{2}$$

SO_2_ conversion can be directly calculated by equation (3) from the difference between inlet and outlet SO_2_ volumetric flows:

(3) $$C_{\text{SO}2}=\frac{V_{{\text{SO}2}_{in}}-V_{{\text{SO}2}_{out}}}{V_{{\text{SO}2}_{in}}}\times 100$$

Sulfur selectivity can be obtained via equation (4) using the amount of product gases detected by MS.

(4) $$S_{solid}=\frac{SO_{2}\;Converted-\sum Product\left(H_{2}S+\mathrm{COS}+{2CS}_{2}\right)}{SO_{2}\;Converted}\times 100$$

**Fig. 2:**
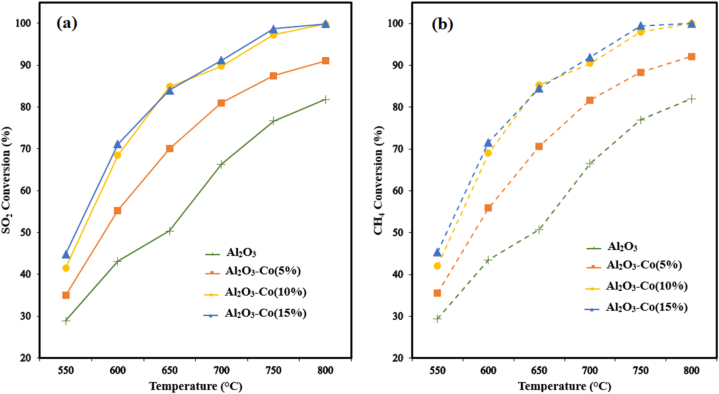
SO_2_ (a) and CH_4_ (b) conversion plots as a function of temperature for different catalysts (Feed compositions = 2 % SO_2_, 1 % CH_4_, 97%Ar; GHSV = 8000 h^−1^).

SO_2_ and CH_4_ conversion plots for all Al_2_O_3_–Co catalysts versus the reactor temperature are given in Fig. 2.

**Fig. 3:**
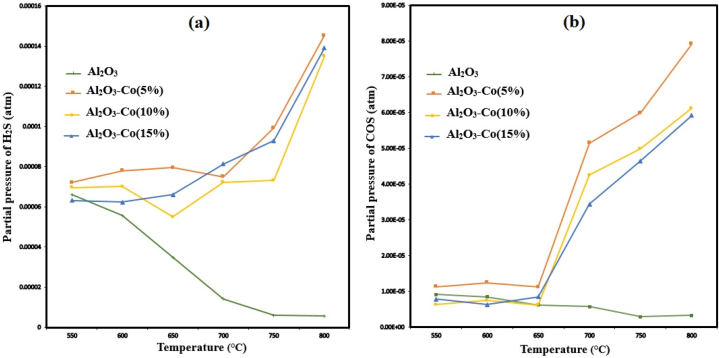
Partial pressures of H_2_S (a) and COS (b) versus temperature for different catalysts (Feed compositions = 2 % SO_2_, 1 % CH_4,_ 97%Ar; GHSV = 8000 h^−1^).

According to Fig. 2a and b, it is obvious that all cobalt containing catalysts exhibited superior catalytic performance than the bare alumina. It should be noted that, because of the high sulfur selectivity and negligible undesired products (Fig. 3a and b), both conversion plots of SO2 and CH4 are almost similar (e.g. for Al2O3–Co(15 %) at 800 °C SO2 conversion is 99.9 % but CH4 conversion is 100 %). As can be observed in Fig. 2a, at lower temperatures (e.g., 550 °C) all the catalysts show low catalytic activities (lower than 50 %).

However, at higher temperatures especially more than 700 °C, Al_2_O_3_–Co(10 %) and Al_2_O_3_–Co(15 %) catalysts show SO_2_ conversions more than 90 % reaching 99.9 % at 800 °C. Also, Al_2_O_3_–Co(5 %) revealed a better performance with SO_2_ conversion of 91 % rather than alumina with SO_2_ conversion of 81 % at 800 °C.

The partial pressure curves of H_2_S and COS by-products against temperature for all the catalysts are compared in Fig. 3a and b, respectively. Considering Fig. 3a, two different trends are obvious within H_2_S plots. One belongs to the bare alumina which produces H_2_S decreased by increasing the temperature, and the other belongs to Al_2_O_3_–Co catalysts, producing H_2_S which is increased slightly with temperature. For alumina at lower temperatures, the conversion is incomplete and there is a lot of unreacted CH_4_ and SO_2_. The unreacted CH_4_ can be decomposed according to equation (5):

(5) $${\text{CH}}_{4}\to \;C+2H_{2}$$

Subsequently produced H_2_ can react with SO_2_ to form H_2_S and water in equation (6):

(6) $$3H_{2}+{\text{SO}}_{2}\to \;H_{2}S+2H_{2}O$$

Given that almost no H_2_ was detected at the reactor outlet and H_2_S is decreasing with increasing the conversion rate, this possibility was confirmed [20]. But for Al_2_O_3_–Co catalysts, seemingly, CS_2_ production increases with increasing the temperature through equation (7) [25]. Then, the produced CS_2_ can react with H_2_O to form H_2_S and COS according to equation (8). Also, CS_2_ could react with CO_2_ to produce COS via equation (9) (Fig. 3b). In the next step, COS reacts with water (product of equation (1)) in equation (10). In addition, produced H_2_ from equation (7) could react with SO_2_ and sulfur (product of equation (1)) through equations (6), (11), respectively, to increase H_2_S production (Fig. 3a). Taking into account H_2_S to COS molar ratios for Al_2_O_3_–Co catalysts at temperatures higher than 700 ᵒC, equation (6) should be the prevailing possibility concurrent with the fact that equation (11) can favor the backward direction as an equilibrium reaction [42,43].

(7) $${\text{CH}}_{4}+S_{2}\to \;{\text{CS}}_{2}+2H_{2}$$

(8) $${\text{CS}}_{2}+H_{2}O\;\to \;\mathrm{COS}+H_{2}S$$

(9) $${\text{CS}}_{2}+{\text{CO}}_{2}\to \;2\;\mathrm{COS}$$

(10) $$\mathrm{COS}+H_{2}O\to \;{\text{CO}}_{2}+H_{2}S$$

(11) $$2H_{2}+S_{2}\to \;2H_{2}S$$

Moreover, the amount of COS increased sharply for all Al_2_O_3_–Co catalysts at higher temperatures, as explained before (according to equations (7)–(9)). Among various reaction pathways, considering H_2_S and COS by-products from Fig. 3a and b, besides more produced COS for Al_2_O_3_–Co (5 %) than Al_2_O_3_–Co (10 %) and Al_2_O_3_–Co(15 %) due to more unreacted SO_2_ and CH_4_, the explained mechanism is more probable. Also, no traces of CS_2_ were observed as by-product in the experiments. It is worth noting that the total amount of H_2_S and COS is negligible with a sulfur selectivity more than 98.8 % at 800 °C for Al_2_O_3_–Co (5 %) showing the lowest activity between synthesized catalysts.

**Fig. 4:**
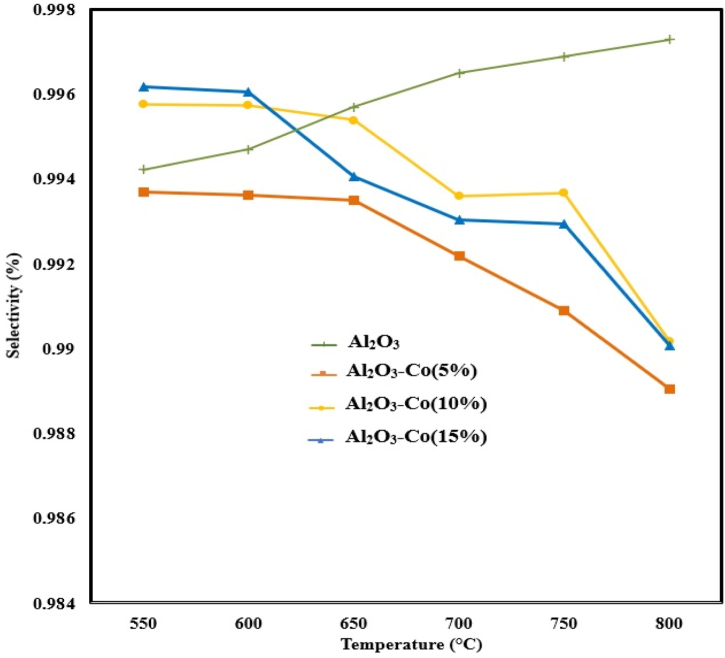
Effect of temperature on sulfur selectivity for the prepared catalysts (Feed compositions = 2 % SO_2_, 1 % CH_4,_ 97%Ar; GHSV = 8000 h^−1^).

In general, all the catalysts prepared demonstrated a high sulfur selectivity exceeding 98.8 % across the range of operating temperatures, as depicted in Fig. 4. However, the sulfur selectivity of the Al_2_O_3_–Co catalysts exhibited a declining trend with increasing reaction temperature. This can be attributed to the fact that the Al_2_O_3_–Co catalysts not only facilitate equation (1) but also accelerate potential side reactions, such as equation (7). According to the data presented in Fig. 4, the disparity in sulfur selectivity between Al_2_O_3_–Co (10 %) and Al_2_O_3_–Co (15 %) was found to be less than 0.1 % at 700 and 750 °C. Moreover, both Al_2_O_3_–Co (10 %) and Al_2_O_3_–Co (15 %) achieved a sulfur selectivity of 99 % at 800 °C. Considering the minimal difference in sulfur selectivity and the superior conversion of SO_2_ for Al_2_O_3_–Co (15 %), it was selected for further investigation of other influential parameters on the reduction of SO_2_ by CH_4_. Although Al_2_O_3_–Co (15 %) exhibited similar catalytic performance at 750 and 800 °C, a temperature of 750 °C was chosen as the primary operating temperature.

The cobalt-alumina catalyst investigated in this study exhibited excellent performance for the reduction of sulfur dioxide to methane. Specifically, the Al_2_O_3_–Co(15 %) catalyst demonstrated superior results, achieving a remarkable SO_2_ conversion rate of 99.3 % and a sulfur selectivity exceeding 99 % at an operating temperature of 750 °C. Notably, this catalyst outperformed the molybdenum-alumina catalyst investigated in our previous research (SO_2_ conversion of 85.8 % and sulfur selectivity of 99.2 %) [38]. However, in comparison to our other study involving a nickel-alumina catalyst [37], the performances of the two catalysts were similar, with only a slightly lower stability observed for the cobalt-alumina catalyst. Moreover, when compared to cerium-based catalysts (both binary and ternary ceria-based catalysts) [24,26,27], the cobalt-alumina catalyst demonstrated superior performance, achieving a 100 % SO_2_ conversion and an 83 % sulfur yield, while also being more cost-effective.

#### 3.2.2 Effect of SO_2_/CH_4_ molar ratio on SO_2_ reduction

**Fig. 5:**
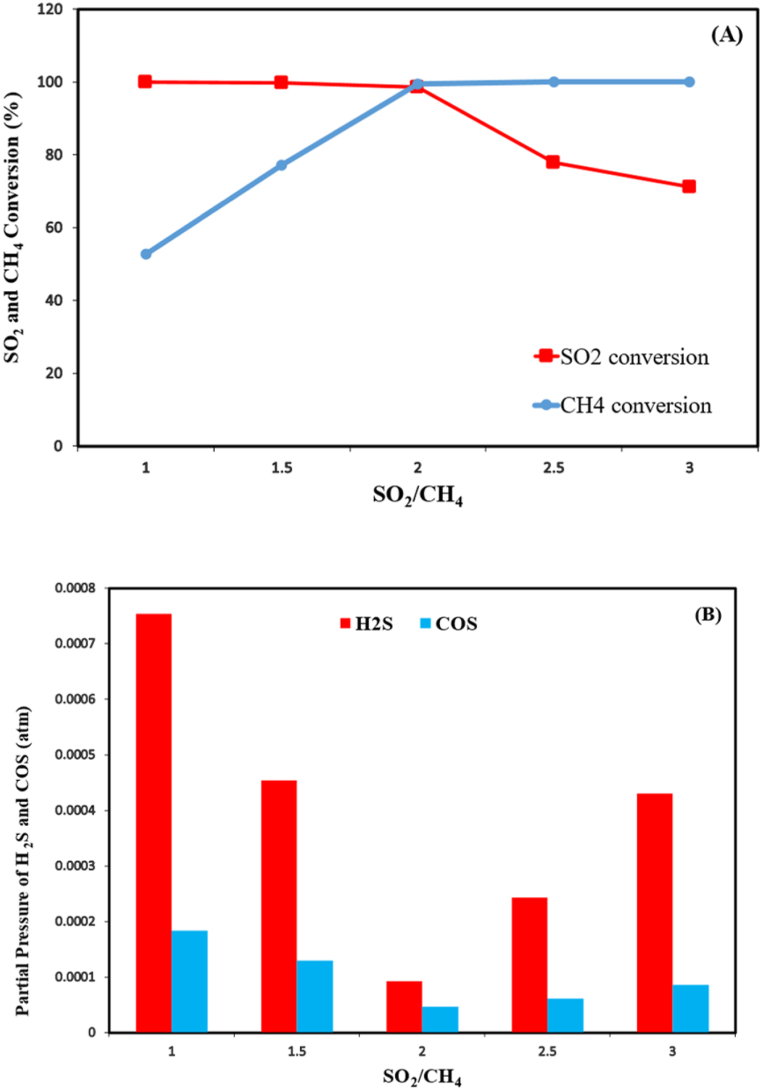
Effects of feed gas composition on SO_2_ conversion (a) and H_2_S, COS production (b) for Al_2_O_3_–Co (15 %) catalyst)GHSV = 8000 h^−1^; temperature = 750 °C).

The effect of changing SO_2_/CH_4_ molar ratio in the feed gas on SO_2_ and CH_4_ conversions as well as H_2_S and COS production rates are given in Fig. 5a and b, respectively.

From Fig. 5 it can be deduced that higher SO_2_ conversions are achieved at SO_2_/CH_4_ molar ratios between 1 and 2, when CH_4_ is in excess with regards to equation (1). On the contrary, when SO_2_ is in excess, its conversion rate drastically reduces to 78 % and 71.25 % in SO_2_/CH_4_ ratios of 2.5 and 3, respectively.

In this condition, because of surplus SO_2_, it seems that H_2_S production mechanism explained in the previous section is boosted leading to more H_2_S and COS production than the condition with stoichiometric ratio of 2. When CH_4_ is in excess, H_2_S production is much higher indicating the major side reaction between SO_2_ and CH_4_ is promoted according to equation (2).

#### 3.2.3 Effect of GHSV on SO_2_ reduction

**Fig. 6:**
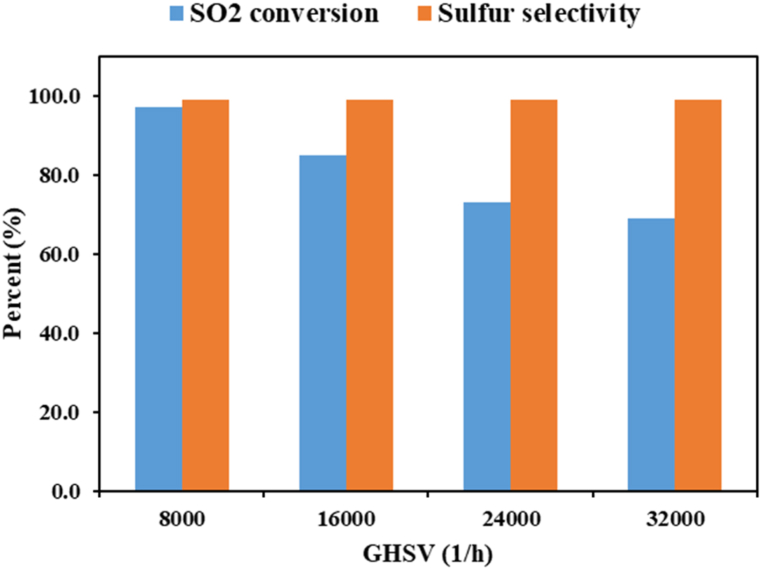
Effect of GHSV on SO_2_ conversion and selectivity for Al_2_O_3_–Co(15 %) catalyst (2 % SO_2_–1% CH_4_–97%Ar at 750 °C).

The effect of GHSV on SO_2_ reduction at 750 °C is shown in Fig. 6. When GHSV of the feed gas increased from 8000 to 32000 1/h, SO_2_ conversion decreased from 97.3 to 69 %. H_2_S and COS partial pressures showed no significant change, and sulfur selectivity remained high in the same value. It can be inferred that lowering contact time between the reactants and the catalyst bed resulted in reducing the conversion rate, expectedly. Also, catalyst selectivity was not a function of GHSV.

#### 3.2.4 On stream stability test of Al_2_O_3_–Co (15 %) catalyst

**Fig. 7:**
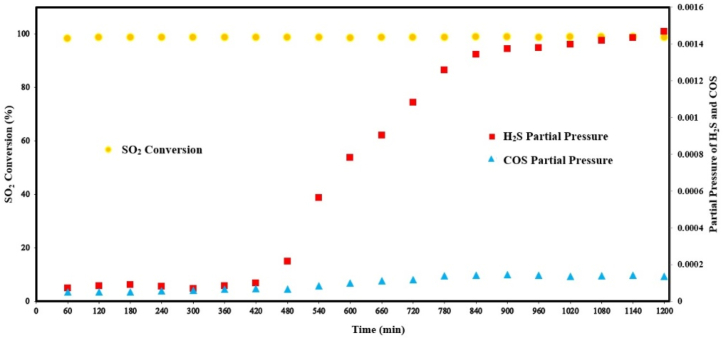
Effect of reaction time on SO_2_ conversion, COS production and H_2_S production over Al_2_O_3_–Co(15 %) catalyst (Feed compositions = 2 % SO_2_,1 % CH_4_, 97%Ar; GHSV = 8000 h^−1^; temperature = 750 °C.

On stream stability of Al_2_O_3_–Co (15 %), as the best catalyst, was examined at 750 °C for 20 h (see Fig. 7). According to Fig. 7, the conversion rate of SO_2_ not only remained high with the time, but also increased about 0.3 % after 20 h.

In the first 420 min, SO_2_ conversion, together with H_2_S and COS production showed no significant change. After that, to the end of the test, SO_2_ conversion started to increase (from 97.3 % at the beginning to 97.6 % at the end) with a smooth slop same as COS, besides a considerable increase for H_2_S. It seems that the major SO_2_ reduction mechanism had a little change. In this regard, it might be concluded that a part of sulfur product from equation (1) reacts with Co_3_O_4_ species to form cobalt sulfides and promote equation (7). Subsequently, more H_2_S is produced and more SO_2_ would be converted through equation (6), according to the mechanism explained for Fig. 3a. However, the presence of two strong reducing agents, such as H_2_ and CH_4_, at the reactor atmosphere could reduce Co_3_O_4_ to CoO and Co_2_C with different catalytic characteristics. It should be considered that H_2_S can react with SO_2_ according to equation (12) to produce sulfur (via Claus reaction) which is another reason for the small increase in SO_2_ conversion. The sulfur selectivity decreased from 99 % at the onset of the process to 92 % after 20 h, resulting in a corresponding reduction in sulfur yield. This suggests that the incorporation of an alternative active species alongside Co_3_O_4_, which could enhance resistance to sulfidation, may be a viable approach to maintaining the sulfur selectivity at its initial level. In a previous investigation involving the reduction of SO_2_ with CH_4_ using MoO_3_-γAl_2_O_3_ catalysts [38], it was observed that the MoO_3_ species transformed into MoS_2_ within a short period. Subsequently, the generated MoS_2_ species exhibited consistent catalytic performance, yielding higher amounts of sulfur compared to MoO_3_. However, the highest achieved SO_2_ conversion rate was approximately 90 % for MoS_2_-γAl_2_O_3_ catalysts.

(12) $${\text{SO}}_{2}+2H_{2}S\;\to \;1.5S_{2}+2H_{2}O$$

An important advantage of SCR of SO_2_ with CH_4_, is its water vapor production according to equation (1), as an active agent for coke removal. With regard to equation (1), 1 mol of water vapor is produced per each mole of consumed SO_2_. Consequently, with the online presence of water vapor, coke deposition would be strongly deferred through equation (13) [44]:

(13) $$C+H_{2}O\;\to \;\text{CO}+H_{2}$$

To investigate the structural changes of the used catalyst during the on-stream stability test, an XRD graph of the used catalyst after 20 h is given in Fig. S4. Moreover, the catalyst was analyzed using a TPO test by Thermo-Gravimeter device in an air environment. Also, the output of the TPO test chamber was connected to the mass spectrometer for detecting oxidation product gases (TG/MS analysis). According to Fig. S4 and the dominant peaks, it is obvious that major species (except alumina support peaks) are Co_3_O_4_, CoS and CoS_2_. Furthermore, reduction originality of H_2_ and CH_4_ resulted in forming CoO, Co_2_C and Co_3_C at small amounts.

**Fig. 8:**
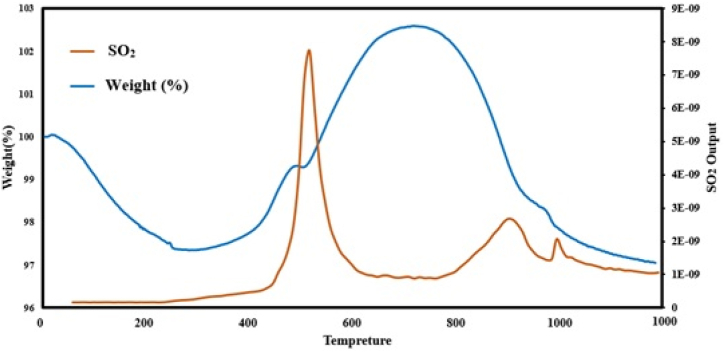
Changes of catalyst weight and SO_2_ MS peaks obtained from Thermo-Gravimeter analysis for the used Al_2_O_3_–Co(15 %) catalyst in 20 h stability test.

Changes of the catalyst weight and MS peaks of SO_2_ obtained from the TG/MS analysis are indicated in Fig. 8. Herein, the temperature was increased to 1000 °C with a constant rate of 30 °C/min, and then, for a few minutes remained constant at 1000 °C.

According to Fig. 8, at first some weight loss appeared due to water desorption from the catalyst surface. At temperatures about 300 °C, equations (14), (15) were accelerated with different effects, equation (14) produced SO_2_ with reducing the catalyst weight, but equation (15) increased the weight of the catalyst producing CoSO_4_ [43]. At temperatures about 500 °C, almost all of the accessible CoS_2_ were oxidized while it took time for all of the accessible CoS to be sulfated until 700 °C, while building a non-diffusible solid shell against oxygen intra diffusion. Subsequently, at 720 °C and close to melting point of CoSO_4_, crystals of this species started melting and vaporizing to open new pathways for O_2_ to access the remaining CoS and CoS_2_. Herein, rates of equations (14), (16), (17) elevated beside melting cobalt sulfates which showed a steeper descending trend versus ascending trend of the catalyst weight between 300 and 700 °C. At 900 °C, at the same time with the second SO_2_ peak and boiling point of Co_3_O_4_, these species started to vaporize and follow reverse direction of equation (17) to a more stable condition [45,46]. The latter led to the condensation of CoO species preventing penetration of oxygen, and finally lowering the rates of equations (14), (16).

Variations in the slops of the catalyst weight loss in Fig. 8 were another sign of this possibility. Finally, approaching 1000 °C, perhaps due to increasing diffusion coefficients and reaction rates, helped oxygen to reach remaining cobalt sulfide species to be oxidized and release SO_2_ forming its third peak in Fig. 8.

(14) $$3\;{\mathrm{CoS}}_{2}+8O_{2}\;\to \;{\text{Co}}_{3}O_{4}+6{\text{SO}}_{2}$$

(15) $$\mathrm{CoS}+2O_{2}\;\to \;{\text{CoSO}}_{4}$$

(16) $$\mathrm{CoS}+\frac{3}{2}\;O_{2}\;\to \;\text{CoO}+{\text{SO}}_{2}$$

(17) $$3\text{CoO}+\frac{1}{2}\;O_{2}\;\to \;{\text{Co}}_{3}O_{4}$$

The FE-SEM image of the catalyst after the stability test does not reveal any discernible structural changes. The XRD graph and TG/MS analysis of the utilized catalyst indicate that two distinct types of changes occur over prolonged periods of time in the reactor. Firstly, a minimal amount of Co_2_C and Co_3_C is formed due to the presence of carbon released from methane (equation (5)). Secondly, CoS and CoS_2_ are formed as a result of sulfur presence. Among these factors, the latter occurs more frequently and primarily contributes to the catalyst's performance alteration (likely due to substantial sulfur accumulation at the catalyst level). Consequently, the catalytic activity undergoes changes over time, leading to an undesirable increase in the production of improper H_2_S by-products. The results demonstrate that the catalytic activity of Co_3_O_4_ alone is significantly superior to its sulfide or carbide forms. To enhance the catalyst's lifespan, the utilization of oxide stabilizers capable of maintaining the stability of cobalt oxide for extended periods is recommended. Additionally, the findings suggest that regenerating the catalyst by subjecting it to high-temperature airflow to convert it back to its oxide form is appropriate.

### 3.3 Determining activation energy

Activation energy was determined by assuming the Arrhenius model for the reaction constants [47].

(18) $$\ln\left(k_{i}\right)=\ln\left(A_{i}\right)-\frac{E_{i}}{kT}$$

In equation (18), k = 8.6173324 × 10^5 eV/K is Boltzmann constant while *T* and $A_{i}$ are absolute temperature and pre-exponential factor, respectively. In Fig. S5, Arrhenius plot for Al_2_O_3_–Co(15 %) catalyst obtained from SO_2_ conversion is presented. The results showed that the amount of activation energy for this catalyst in the temperature range of 550–800 °C is 0.228 eV. It is worth noting that in our previous study calculated activation energy for Al_2_O_3_–Mo10 (as the best catalyst) was 0.33 eV in a same operating condition [46]. Also, Guiance et al. [47] reported this parameter 0.57 eV for Al_2_O_3_–Cr_2_O_3_ in a temperature range of 25–45 °C. The superior performance of Al_2_O_3_–Co(15 %) rather than Al_2_O_3_–Mo10 is consistent with its lower activation energy.

## 4 Conclusion

To address the issue of SO_2_ emissions, selective catalytic reduction of SO_2_ with CH_4_ using cobalt alumina catalysts was investigated as a sustainable solution. Cobalt oxide species were successfully introduced onto the alumina support using the wet impregnation technique. All the cobalt-containing catalysts outperformed bare alumina in the process. The Al_2_O_3_–Co(15 %) catalyst exhibited superior results, with a SO_2_ conversion of 99.3 % and sulfur selectivity of over 99 % at 750 °C. By varying the SO_2_/CH_4_ molar feed ratios from 1 to 3, it was concluded that the best performance was achieved at the stoichiometric ratio of SO_2_/CH_4_ = 2.

The 20 h on-stream stability test of Al_2_O_3_–Co(15 %) revealed a decrease in sulfur selectivity from 99 % to 92 %, while SO_2_ conversion remained high. The structural changes in the catalyst after the on-stream stability test were evaluated using X-ray diffraction (XRD) analysis and temperature-programmed oxidation (TPO) experiments. The XRD pattern indicated the presence of Co_3_O_4_, CoS, and CoS_2_ as major components, while the amounts of CoO, Co_2_C, and Co_3_C species were not significant. It was concluded that a different active species is needed beside Co_3_O_4_ for improving the resistance to sulfidation, in terms of the long-term industrial processes. Additionally, the activation energy of the Arrhenius model for the Al_2_O_3_–Co(15 %) catalyst was calculated at 0.228 eV within the temperature range of 550–800 °C. These findings further proved the potential of the catalyst for industrial applications.

## Data availability statement

Data generated and utilized for analyses of results presented in this manuscript are available from the corresponding authors on reasonable requests.

## CRediT authorship contribution statement

**Masoud Khani:** Conceptualization, Data curation, Formal analysis, Investigation, Methodology, Resources, Software, Validation, Visualization, Writing – original draft, Writing – review & editing. **Seyyed Ebrahim Mousavi:** Conceptualization, Data curation, Formal analysis, Investigation, Methodology, Resources, Software, Validation, Visualization, Writing – original draft, Writing – review & editing. **Reza Khalighi:** Writing – review & editing, Writing – original draft, Methodology, Conceptualization, Data curation, Formal analysis, Investigation, Resources, Software, Validation, Visualization. **Saeed Abbasizadeh:** Conceptualization, Data curation, Formal analysis, Investigation, Methodology, Resources, Software, Validation, Visualization, Writing – original draft, Writing – review & editing. **Hassan Pahlavanzadeh:** Conceptualization, Funding acquisition, Project administration, Supervision. **Habib Ale Ebrahim:** Conceptualization, Funding acquisition, Project administration, Supervision. **Abbas Mozaffari:** Conceptualization, Funding acquisition, Project administration, Supervision.

## Declaration of competing interest

The authors declare that they have no known competing financial interests or personal relationships that could have appeared to influence the work reported in this paper.
