# Supplementary material for: Cobalt oxide-alumina catalysts for the methane-assisted selective catalytic reduction of SO2 to sulfur
Source: Heliyon. 2023 Oct 27;9(11):e21269. doi: 10.1016/j.heliyon.2023.e21269 (PMC10632700; doi:10.1016/j.heliyon.2023.e21269)
Supplement: Multimedia component 1 [file mmc1.docx]

**Cobalt oxide-alumina catalysts for the methane-assisted selective catalytic reduction of SO_2_ to sulfur**

Masoud Khani ^a^, Seyyed Ebrahim Mousavi ^b^[[1]](#footnote-1)^*^, Reza Khalighi ^c^[[2]](#footnote-2)^*^, Saeed Abbasizadeh ^b^, Hassan Pahlavanzadeh ^b^, Habib Ale Ebrahim ^a^, Abbas Mozaffari ^d^

**^a^** Faculty of Chemical Engineering, Petrochemical center of Excellency, Amirkabir University of Technology, Tehran, Iran

**^b^** Faculty of Chemical Engineering, Tarbiat Modares University, Tehran, Iran

**^c^** School of Chemical Engineering, College of Engineering, University of Tehran, Tehran, Iran

**^d^** Research and Development Unit, Sarcheshmeh Copper Complex, Kerman, Iran

*Corresponding author E-mail address:

s.ebrahimmousavi66@gmail.com (Seyyed Ebrahim Mousavi**)**

[rezakhalighii@gmail.com](mailto:rezakhalighii@gmail.com) (Reza Khalighi)

******************************************************************

**Content:**

- Figure S1
- Figure S2
- Figure S3
- Figure S4
- Figure S5
- Table S1


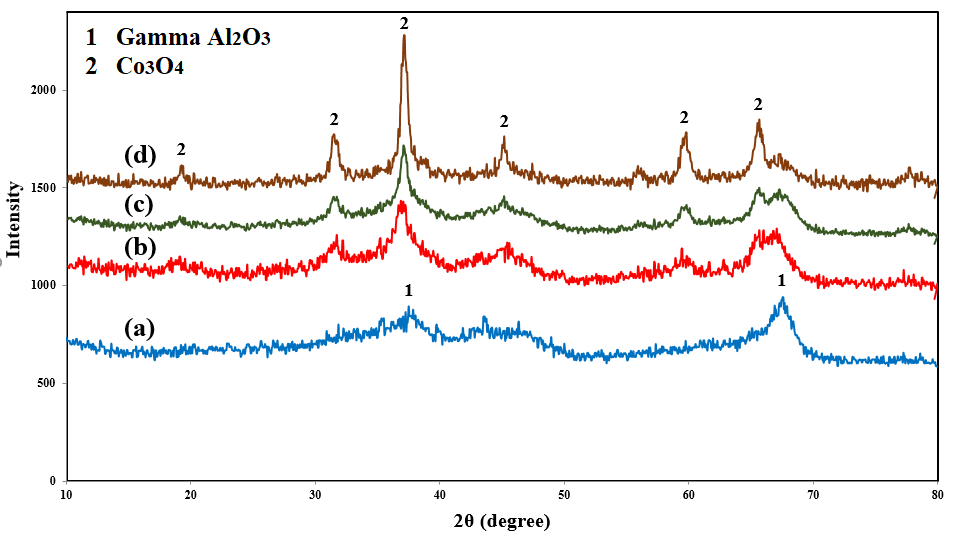


**Fig. S1.** XRD patterns of the alumina (a) Al_2_O_3_-Co(5%) (b) Al_2_O_3_-Co (10%) (c) and Al_2_O_3_-Co(15%) (d).


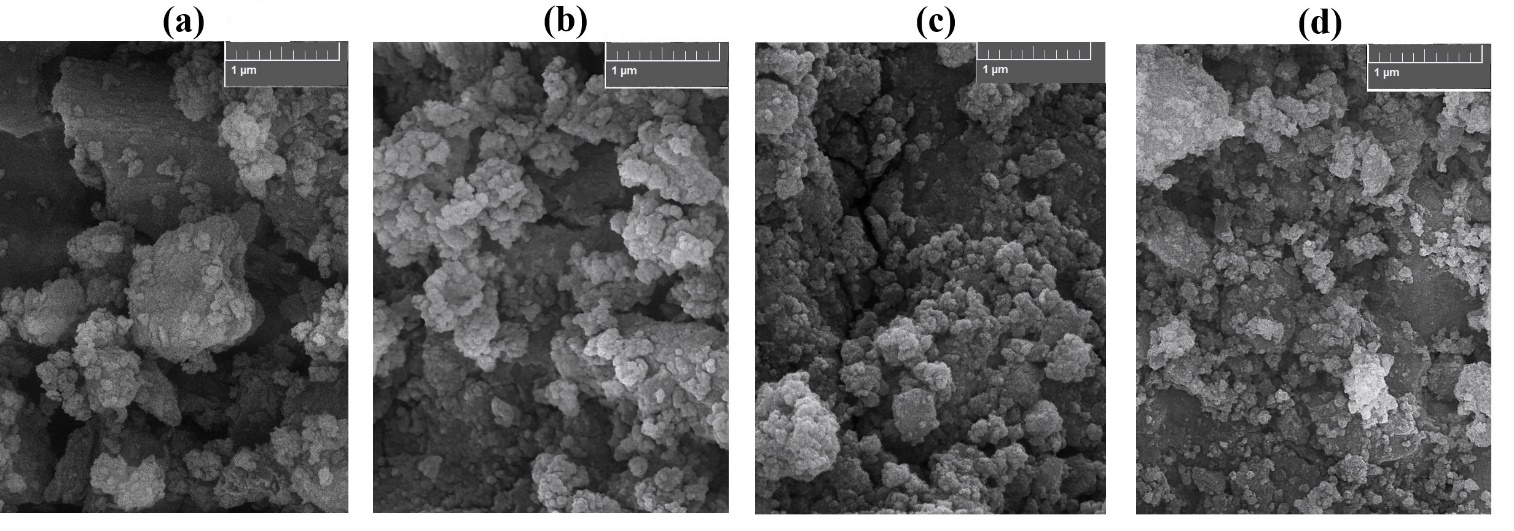


**Fig. S2.** FE-SEM images of Al_2_O_3_ (a) Al_2_O_3_-Co(5%) (b) Al_2_O_3_-Co (10%) (c) and Al_2_O_3_-Co(15%) (d).


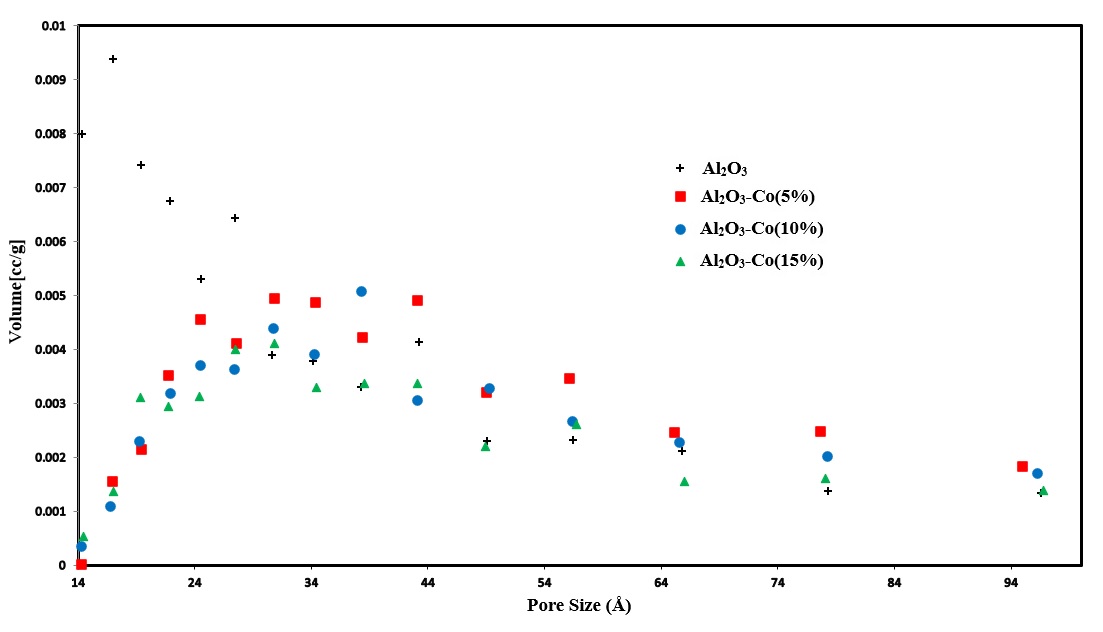


**Fig. S3**. BJH pore size distribution of synthesized catalysts.


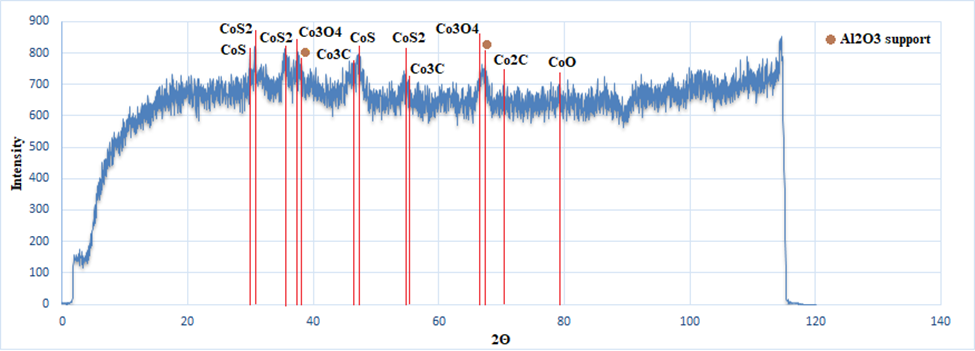


**Fig. S4.** XRD graph of used catalyst during stability test of 20 hours


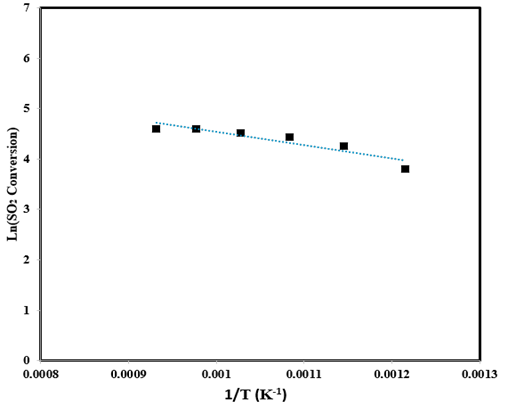


**Fig. S5.** Arrhenius plots for SO_2_ reactions with CH_4_ for Al_2_O_3_-Co(15%) catalysts

**Table S1.** BET surface area, total pore volume, average pore diameter and XRF results of all catalysts.

| Sample | S_BET_ (m^2^/g) | Average pore diameter (Å) | V_total_ (pore volume) (cm^3^/g) | Co_3_O_4_ %  (Via XRF) |
| --- | --- | --- | --- | --- |
| **Al_2_O_3_** | **347.1** | **43.62** | **0.3786** | **-** |
| **Al_2_O_3_-Co(5%)** | **228.3** | **63.44** | **0.3659** | **5.1** |
| **Al_2_O_3_-Co(10%)** | **212.4** | **66.32** | **0.3479** | **9.9** |
| **Al_2_O_3_-Co(15%)** | **196.4** | **68.95** | **0.3265** | **15.2** |

1. **†*** Corresponding author. [s.ebrahimmousavi66@gmail.com](mailto:s.ebrahimmousavi66@gmail.com) (Seyyed Ebrahim Mousavi). [↑](#footnote-ref-1)
2. **†*** Corresponding author. [rezakhalighii@gmail.com](mailto:rezakhalighii@gmail.com) (Reza Khalighi). [↑](#footnote-ref-2)
